# Sports Level and the Personality of American Football Players in Poland

**DOI:** 10.3390/ijerph182413026

**Published:** 2021-12-10

**Authors:** Paweł Piepiora, Damian Kwiatkowski, Justyna Bagińska, Dimitris Agouridas

**Affiliations:** 1 Faculty of Physical Education and Sport, Wroclaw University of Health and Sport Sciences, 51-612 Wrocław, Poland; damian.kwiatkowski13@gmail.com; 2 Faculty of Economics and Management, University of Business in Wrocław, 53-238 Wrocław, Poland; Justyna.Baginska@handlowa.eu; 3 Faculty of Economics and Administrative Sciences, Alanya HEP University, Alanya 07400, Turkey; dimitrios.agouridos@ahep.edu.tr

**Keywords:** personality, Big Five, American football, sport level, league

## Abstract

Research on personality in sport is very popular as it allows prediction of the behavior of players in the starting situation. Hence, verifications of players due to their sports level may turn out to be crucial. Due to the dynamic development of American football in Poland, we undertook research to verify the relationship between the sports level and the personality of these players. The Big Five personality study that we carried out involved players aged from 20 to 29—the representatives of American football clubs in Poland (*N* = 140) from three league games levels: LFA 1 (*n* = 75), LFA 2 (*n* = 40), and LFA 9 (*n* = 25). The NEO-FFI personality questionnaire was used as a research tool. The players from the top-level games were characterized by their openness to experience, the level of which decreases along with the decrease in the players’ sports levels. The differences in openness to experience were revealed, first of all, in divergent thinking and creativity. It was ascertained that openness to experience is a characteristic personality trait for American football players in Poland. Therefore, systematic conduct of personality tests among American football players in Poland, in the process of selecting candidates for the highest levels of football competition, would be recommended. This might significantly affect the development of the sports level of this discipline in Poland. The obtained results of research on personality may, moreover, prove to be useful in selecting players and improving the predictions of important sports behaviors in American football in Poland.

## 1. Introduction

American football is considered a brutal contact sport with players’ collisions being an integral part of the game [1]. Interestingly enough, it is one of the few team sports in the world that allows for an attack by a player who is present on the pitch, but who is not actually taking an active part in the action. In addition, in comparison to other sports, this discipline is distinguished by above-average explosiveness and the ensuing force used during the game [2]. It should be noted, however, that American football is based on very advanced tactical arrangements. Virtually nothing that happens on the pitch can be a matter of chance. Therefore, an American football player, regardless of the sport level represented, should take care of both his proper motor and mental preparation [3]. Sometimes beginners give up further training precisely because of the lack of appropriate psychological predispositions [4]. Especially in Europe, American football players struggle with many difficulties resulting from their sports discipline in their everyday life [5]. Moreover, they have to show the right mental attitude to training and matches almost round the clock, and learn to deal with chronic pain and injuries, which are permanently inscribed in this sport discipline [6,7]. These factors show how important sport psychology and related science is for American football [8]. In this respect, research on the athletes’ personality is important, as it is determined by the athletes’ environment [9,10]; hence the supposition that personality may depend on the sports level of players. Research in this area is scarce and inconsistent. On the one hand, there are no differences in the personality profiles of athletes in relation to the sport level represented [11,12,13,14]. On the other hand, such differences are present in different dimensions of their personality, depending on the trained sports discipline [15,16,17,18]. However, it is important that sport activity influences the personality of people who train. In the context of athletic performance, personality traits relate to long-term athletic success, interpersonal relationships, and the mental states of athletes before, during, and after competition. In the context of health-related exercises, personality traits refer to the use of leisure time, strength and mobility in old age, as well as unhealthy and addictive physical behaviors [6,8,10,14,16]. So far, no studies have been conducted to verify the relationship between personality and sports experience among American football players in Poland. Therefore, we decided that it is worth examining this relationship.

The first rudimentary information on American football in Poland comes from the 1990s, but the idea of creating a nationwide association of the American football movement in Poland appeared only in May 2004 [19]. Two years later, the Polish American Football League (PLFA) was established. In 2008, the competition of Polish teams was divided into two divisions. Additionally, in 2011, the third league of American football was added. Since 2013, there has been an official Polish American football representation. A further step in the development of this discipline in Poland was the establishment of the professional League of American Football (LFA), a private limited company, in 2017 at three senior levels: First League of American Football (LFA1), Second League of American Football (LFA2), Third League of Nine-men American Football (LFA9), and one junior level: the Junior League of American Football (JLFA) [20]. Currently, there are 32 teams in total in the senior leagues, and 9 teams in the junior games.

Taking the above into consideration, the aim of the present research was to verify the relationship between the sports level and the personality of American football players in Poland. For this purpose, players from all levels of the American Football League senior games existing in Poland were examined. It was decided to verify the following hypothesis: there are differences in the personality profiles of American football players in Poland, depending on the LFA games sports level.

## 2. Materials and Methods

With reference to the ethical approval of the study, it was conducted on the basis of positive opinion no. 20/2019 of the Senate Committee for Scientific Research Ethics at the University School of Physical Education in Wrocław, Poland.

### 2.1. Tested Persons

**Figure 1:**
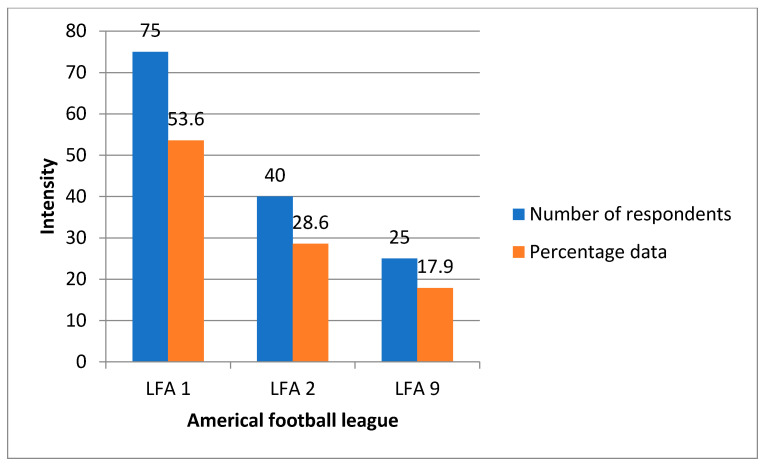
Division of the surveyed players according to the represented LFA game level.

The research sample consisted of players representing 37 American football clubs in Poland, that belonged to LFA, a private limited company, in the 2020 season. These clubs play their games in the following 3 leagues: LFA 1, LFA 2, and LFA 9 (Figure 1). Participation in the study was offered to all players of the above-mentioned leagues, but only 140 footballers expressed a voluntary willingness to participate in the study and only they were included in it. The respondents were men aged from 20 to 29 and were training competitively. The players were characterized by a variety of represented physical conditions, motor skills, education (secondary or higher), and their professional background. The tested players represent all positions in the offensive and defensive formations, and therefore they are subjected to various mental and physical loads, which are characteristic for a given position on the pitch. The respondents constituted a significant cross-section of American football players aged 20–29 years in Poland.

### 2.2. Method

The study used a five-factor model of personality, known as the Big Five [21]. This model is widely recognized as the most reliable and tested theory of personality traits. According to this method, the human personality consists of 5 main characteristics and 30 subordinate ones. Personality traits, according to the above method, are the following:

- Neuroticism (anxiety, aggressive hostility, depression, impulsiveness, hypersensitivity, shyness);
- Extraversion (sociability, cordiality, assertiveness, activity, sensation seeking, positive emotionality);
- Openness to experiences (imagination, aesthetics, feelings, actions, ideas, values);
- Agreeableness (trust, straightforwardness, altruism, compassion, modesty, tendency to be sympathetic);
- Conscientiousness (competence, tendency to order, duty, striving for achievement, self-discipline, prudence).

Upon characterizing the Big Five, one should pay attention to several important aspects of the personality dimensions, namely that these features characterize the “normal personality”, although their extreme intensity may contribute to the development of behavioral disorders and psychosomatic diseases. These features are not characteristic of classical personality types, and the Big Five describes mainly their extreme poles. In fact, these personality traits have a continuous nature and, like other mental properties, have a normal distribution in a population. The Big Five therefore allows a description of each personality. Moreover, these features should not be simply evaluated. A given pole may be associated not only with positive but also negative trends in behavior, both for the social environment and for a given individual. Therefore, one should not make a one-sided assessment of personality, because each of these features has its advantages and disadvantages. Additionally, that is why the Big Five factors meet the criteria required for the characteristics of the basic personality dimensions [21,22].

The research tool was the NEO-FFI Personality Questionnaire, which is widely used in personality research in the field of sports psychology [22]. The questionnaire consisted of 60 self-report statements, the truthfulness of which was assessed by the respondents themselves on a five-point scale: “definitely not”, “rather not”, “I have no opinion”, “rather yes”, “definitely yes”. Due to the fact that the Big Five features contain 12 items each, the raw score for each of the features ranges from 0 to 48 points. The answer key followed a design: the higher numerical score on the scale—the greater intensity of a given feature. Thus, the greater the number of diagnostic responses, the higher the scores on the scale of neuroticism, extraversion, openness to experience, agreeableness, and conscientiousness—and as a result, the greater the neuroticism, extraversion, openness to experience, agreeableness, and conscientiousness are understood as a person’s characteristics. The calculation of the results of each study was performed using the tables of Polish standards for the sex and age (15–19, 20–29, 30–39, 40–49, 50–80) of the respondent. First, the raw scores were summed and then converted to sten scores. The interpretation of the results included two aspects: psychometric and psychological. Psychometric interpretation is related to presenting the results of a given player against the background of the reference group appropriate for him and assumes the necessity of interval estimation of the results. The results ranging from 1 to 3 sten should be treated as low, from 7 to 10 as high, and from 4 to 6 as average. Based on a specific profile, one may make a psychological interpretation of the obtained results.

### 2.3. Procedure

The research was conducted between December 2020 and February 2021. Due to the COVID-19 pandemic and the periodic lockdown in Poland, the research was conducted online. The tests were individual and were time-limited to an hour. The average time for one test was about 15 min. The research was carried out using the CAWI (Computer Assisted Web Interview) method with the use of SURVIO software for conducting surveys. Each of the American football players in Poland belonging to LFA1, LFA2, LFA9, via e-mail, received temporary access to the NEO-FFI questionnaire along with a personal request to complete it. All respondents consented to the processing of the obtained results for the purposes of scientific research. Before commencing the questionnaire, the respondents read the filling instructions.

### 2.4. Statistical Analyses

First, a general personality profile of American football players in Poland was generated. Then, the nature of the distribution of variables in individual groups was assessed, for which the Shapiro–Wilk test was used, and the significance level was set at *p* = 0.05. On the basis of the obtained results, adequate tests were selected for further analysis. In cases where the distribution of results in each of the groups was normal, a one-way analysis of variance ANOVA was performed. However, when the distribution in at least one group differed from the normal distribution, the Kruskal–Wallis test was used.

## 3. Results

Descriptive statistics of the personality profile of American football players aged 20–29 years in Poland were calculated in the first part of result analysis, as presented in Table 1.

On the neuroticism scale, the raw scores (RS) ranged from Min = 5 to Max = 37 with the median Me = 15, and the upper quartile Q75 = 21. The distribution of the results was different from the normal distribution (*p* < 0.001). On the sten scale, the results on neuroticism ranged from Min = 1 to Max = 10. This means that the study group was quite diverse (it included both people with the lowest and the highest possible levels of neuroticism). Yet, the results of three quarters of players did not exceed the level of Q75 = 6. The distribution was different from normal (*p* < 0.001).

The raw scores (RS) on the extraversion scale ranged from Min = 14 to Max = 46, with the median Me = 34, and the upper quartile Q75 = 39. The distribution of the results differed from the normal distribution (*p* < 0.001). On the sten scale, the results on extraversion ranged from Min = 1 to Max = 10. Again, this means that the study group was quite diverse (and included both people with the lowest and the highest possible levels of extraversion). The upper quartile here was Q75 = 9 and again, the distribution was different from normal (*p* < 0.001).

On the scale of openness to experience, raw scores (RS) ranged from Min = 15 to Max = 42, with the median Me = 28, and the upper quartile Q75 = 32.5. The mean raw score for openness to experience was M = 28.17 with standard deviation SD = 5.86. The distribution of the results was normal (*p* > 0.05). On the sten scale, the results regarding openness to experience ranged from Min = 1 to Max = 10. This means that the study group was quite diverse (and included both people with the lowest and the highest possible levels of openness to experience). The upper quartile was Q75 = 7 and the distribution was different from normal (*p* < 0.05).

On the agreeableness scale the raw scores (RS) ranged from Min = 14 to Max = 40, the median was Me = 29, and the upper quartile Q75 = 32. The average for raw scores on the agreeableness scale was M = 28.44, with standard deviation SD = 5.82. The distribution of the results was normal (*p* > 0.05). On the sten scale, the results on agreeableness ranged from Min = 1 to Max = 10. This means that the study group was quite diverse (and included both people with the lowest and the highest possible levels of agreeableness). The upper quartile here was Q75 = 7 and the distribution was different from normal (*p* < 0.05).

On the conscientiousness scale, raw scores (RS) ranged from Min = 15 to Max = 48, with the median Me = 36 and upper quartile Q75 = 41. The distribution of results was different from the normal distribution (*p* < 0.05). On the sten scale, the results on conscientiousness ranged from Min = 1 to Max = 10. Yet, again, the study group turned out to be quite diverse (including both people with the lowest and the highest possible levels of conscientiousness). The upper quartile here was Q75 = 9 and the distribution was different from normal (*p* < 0.001).

In the second step of the research procedure, the personality profiles of the American football players aged 20–29 years in Poland were verified in terms of the games level. The analysis began with the assessment of the variables distribution in individual groups. For this purpose, the Shapiro–Wilk test was used; the level of significance was assumed at *p* = 0.05. Based on its results, it was decided to select the appropriate test for further analysis. One-way ANOVA was performed whenever the distribution of results was normal. On the other hand, when the distribution in at least one group differed from the normal distribution, the Kruskal–Wallis test was used. Table 2 presents the data on the personality profiles of American football players aged 20–29 years in Poland in terms of the LFA game level.

There were no statistically significant differences in the raw scores on the neuroticism scale (*p* > 0.05) between players from different game levels. The result ranges in all groups differed slightly, as follows: among LFA 1 players, the results ranged from Min = 5 to Max = 36, with the lower quartile Q25 = 12, median Me = 15, and upper quartile Q75 = 21. Among LFA 2 players, the results ranged from Min = 7 to Max = 37, with the lower quartile Q25 = 11, median Me = 15, and upper quartile Q75 = 19. Among LFA 9 players, the results ranged from Min = 6 to Max = 34, with the lower quartile Q25 = 10, the median Me = 14 and the upper quartile Q75 = 21. There were no statistically significant differences in the sten scores on the neuroticism scale (*p* > 0.05) between players from different game levels. The ranges of results in all groups were comparable, as follows: among LFA 1 players the results ranged from Min = 1 to Max = 10, with the lower quartile Q25 = 3, median Me = 4, and the upper quartile Q75 = 6. Among the LFA 2 players, the results ranged from Min = 2 to Max = 10, lower quartile Q25 = 3, median Me = 4, and upper quartile Q75 = 6. Among LFA 9 players, the results ranged from Min = 1 to Max = 9, with the lower quartile Q25 = 2, the median Me = 4, and the upper quartile Q75 = 6.

There were no statistically significant differences between the different game levels players regarding the raw scores on the extraversion scale (*p* > 0.05). The ranges of results in all groups did not differ much, as follows: among LFA 1 players, the results ranged from Min = 17 to Max = 45, with the lower quartile Q25 = 30, median Me = 35, and the upper quartile Q75 = 40. Among LFA 2 players, the results ranged from Min = 15 to Max = 46, with the lower quartile Q25 = 30, median Me = 34, and upper quartile Q75 = 38. Among LFA 9 players, the results ranged from Min = 14 to Max = 44, lower quartile Q25 = 26, median Me = 33, and upper quartile Q75 = 37. There were no statistically significant differences between players of different game levels in terms of sten scores on the extraversion scale (*p* > 0.05). The ranges of results in all groups were similar: among LFA 1 players, the results ranged from Min = 2 to Max = 10, with the lower quartile Q25 = 6, median Me = 8, and the upper quartile Q75 = 10. Among LFA 2 players, the results ranged from Min = 2 to Max = 10, with the lower quartile Q25 = 7, median Me = 8, and the upper quartile Q75 = 9. Among LFA 9 players, the results ranged from Min = 1 to Max = 10, with the lower quartile Q25 = 5, median Me = 7, and the upper quartile Q75 = 9.

However, there were statistically significant differences between the different game levels players in the raw scores on the openness to experience scale (F = 3.080; df1 = 2; df2 = 137; *p* < 0.05). In the case of LFA 1 players, the results ranged from Min = 18 to Max = 42, with the median Me = 29. The mean score for raw scores in the openness to experience scale was M = 29.29, with the standard deviation SD = 5.45. In the case of LFA 2 players, the results were lower and ranged from Min = 17 to Max = 42, with the median Me = 27. The mean score for raw scores in the openness to experience scale was M = 27.03 with standard deviation SD = 5.56. LFA 9 players also had lower scores—they ranged from Min = 15 to Max = 40, with the median Me = 24. The mean raw score on the openness to experience scale was M = 26.64 with standard deviation SD = 6.96. There were no statistically significant differences in the openness to experience scale between different game levels players (*p* > 0.05). The ranges of results in all groups differed slightly, as follows: among LFA 1 players, the results ranged from Min = 3 to Max = 10, with the lower quartile Q25 = 5, median Me = 6, and the upper quartile Q75 = 7. Among LFA 2 players, the results ranged from Min = 2 to Max = 9, with the lower quartile Q25 = 4, median Me = 6, and the upper quartile Q75 = 6. Among LFA 9 players, the results ranged from Min = 1 to Max = 10, with the lower quartile Q25 = 3, median Me = 5, and the upper quartile Q75 = 7.

There were no statistically significant differences between the different game levels players in the agreeableness scale (F = 2.417; df1 = 2; df2 = 137; *p* > 0.05). The ranges of scores in all groups were similar, as follows: among LFA 1 players, the scores ranged from Min = 14 to Max = 39, with the median Me = 28. The mean score for the raw scores on the agreeableness scale was M = 27.44 with standard deviation SD = 5.77. Among LFA 2 players, the results ranged from Min = 16 to Max = 39, with the median Me = 29. The mean score for raw scores on the agreeableness scale was M = 29.53 with standard deviation SD = 5.60. Among the LFA 9 players, the results ranged from Min = 19 to Max = 40, and the median was Me = 31. The mean score for raw scores on the agreeableness scale was M = 29.68 with standard deviation SD = 6.00. There were no statistically significant differences in the sten scores on the agreeableness scale (*p* > 0.05) between the different game levels players. The ranges of results in all groups were slightly different; however, among LFA 1 players, the results ranged from Min = 1 to Max = 10, with the lower quartile Q25 = 4, median Me = 5, and the upper quartile Q75 = 7. Among LFA 2 players, the results ranged from Min = 2 to Max = 10, with the lower quartile Q25 = 5, median Me = 6, and the upper quartile Q75 = 8. Among LFA 9 players, the results ranged from Min = 3 to Max = 10, with the lower quartile Q25 = 4, median Me = 6, and the upper quartile Q75 = 8.

There were no statistically significant differences between the different game level players regarding the raw scores on the conscientiousness scale (*p* > 0.05). The range of results in all groups was slightly different, as follows: among LFA 1 players the results ranged from Min = 17 to Max = 47, with the lower quartile Q25 = 30, median Me = 37, and the upper quartile Q75 = 41. Among LFA 2 players, the results ranged from Min = 15 to Max = 46, with the lower quartile Q25 = 28, median Me = 34, and the upper quartile Q75 = 40. Among LFA 9 players, the results ranged from Min = 16 to Max = 48, with the lower quartile Q25 = 30, median Me = 36, and the upper quartile Q75 = 40. There were no statistically significant differences in the sten scores on the conscientiousness scale between different game levels players (*p* > 0.05). The ranges of results in all groups were comparable, as follows: among LFA 1 players, the results ranged from Min = 2 to Max = 10, with the lower quartile Q25 = 6, median Me = 8, and the upper quartile Q75 = 9. Among LFA 2 and LFA 9 players, the results ranged from Min = 1 to Max = 10, with the lower quartile Q25 = 5, median Me = 7, and the upper quartile Q75 = 9.

## 4. Discussion

In our study, we managed to reach the research goal, which was to verify the relationship between the sports level and the personality of American football players in Poland. The research hypothesis has been verified positively—there are differences in the personality profiles of American football players in Poland depending on the sports level of the LFA games. It was first necessary to determine what level of sports advancement each person from the research group represented. This assessment was carried out subjectively on the basis of many years of observation of American football in Poland, as well as excellent knowledge of the discipline environment. The LFA 1 League, being the highest class of the game in Poland (as of late 2020), brings together by far the best teams, whose players, compared with LFA 2 and LFA 9, were characterized by significantly better motor, technical, and tactical preparation. It should be noted that in the event of creating the Polish national team, the players of the three best LFA 1 teams in the 2020 season would be the backbone of the list appointed to represent the Polish national colors. In recent years, the LFA 2 has had a much lower overall sports level than the LFA 1. Despite the fact that in the second league there were some above average players, the average level of football and athletic advancement was visibly lower. LFA 9, on the other hand, is a league that exists for newly established and developing teams that are not able to gather the full team needed to play 11-person football, or teams that do not have enough capital to cope with this challenge. On average, this league includes players whose football and motor advancement is definitely weaker than at the first two game levels in Poland. It should be noted that, in the event of promotion or transfer to a higher league of American football, the club that was a newcomer in the first year of games, most often visibly deviated from the overall level, to the detriment of rivals from a given league. A similar situation occurred with the relegation, where the club joining the league definitely stood out from the rest. Based on the analysis of the conducted research results, there exist statistically significant differences in the raw scores on the openness to experience scale (F = 3.080; df1 = 2; df2 = 137; *p* < 0.05) between players of different game levels. In the case of LFA 1 players, the mean score on the openness to experience scale was M = 29.29 with the standard deviation SD = 5.45. In the case of LFA 2 players, the average results were lower and amounted to M = 27.03 with the standard deviation SD = 5.56. However, in the case of LFA 9 players, lower scores were also observed, which gave an overall result of M = 26.64 with a standard deviation of SD = 6.96. This means that the highest game level players are characterized by the greatest openness to experience, the level of which decreases with the sports level. This, in turn, may imply that the sport level represented has an influence on the American football player’s personality in Poland. The research hypothesis has been partially positively verified—there are differences in the personality profiles of American football players in Poland depending on the sports level of the LFA games, namely: the differences exist only in the dimension of openness to experience. It is worth mentioning that people with a higher openness to experience are more interested in both the external and internal world. They show greater creativity and a vivid and creative imagination. In addition, they often feel intellectual curiosity and interest in art. At the same time, they are unconventional, prone to questioning authority, independent in judgment, and focused on discovering new ideas. On the other hand, people with lower openness to experiences are characterized by more conventional behavior, as well as conservatism in views. Recognizing traditional values and having pragmatic interests, they prefer commonly accepted ways of doing things. Hence, the supposition that the differences in openness to experience were revealed primarily in divergent thinking and creativity of American football players in Poland [21,22]. Therefore, in our opinion, American football players in Poland, characterized by the highest level of openness to experience, have the greatest personality potential to achieve championship at the national level in the long run. Moreover, the research results clearly show that the identification of the players’ personality traits, not only in the selection process, is very important. By discovering the features of players, the coaches can significantly improve the process of recruiting potential players, but also, to some extent, minimize the risk of recruitment failure. Moreover, the coaches, knowing the personality traits of their players, are able to influence them during matches more easily and consciously.

However, the important thing is that no statistically significant difference was found in the openness to experience and other personality dimensions as a result of the sten score. This means that there are no differences in the interpretation of American football players personality depending on the sports level. American football players from the studied leagues are characterized by high extraversion and conscientiousness as well as average neuroticism, openness to experience, and agreeableness, i.e., the personality profile of a typical athlete. The obtained results confirm previous studies between the personality and the sports level of Polish volleyball players [23] and emphasize the role of sport in shaping the personality [24]. Sports activity shapes the personality and the shaped personality traits have an impact on taking solutions in the starting situation [25]. It should be associated with the specificity of sports rivalry and slightly different psychological requirements that sports disciplines impose on competitors. Therefore, among American football players in Poland, there was a significant difference in openness to experience, and among Polish volleyball players, there were significant differences in neuroticism, extraversion, and conscientiousness. However, generally, athletes are distinguished from non-training people by high extraversion, which characterizes their social interactions, their dimensions, quality, their level of energy and activity [11,17,26,27,28,29,30,31], and their conscientiousness—a dimension that distinguishes the level of organization, persistence, and motivation of an individual in pursuing a goal [32,33,34]. Additionally, champions are additionally distinguished by low neuroticism, which reflects emotional adjustment versus emotional imbalance [10,13,18,35,36,37,38,39,40,41,42]. Additionally, the dimensions of openness to the experiences (describes the human tendency to seek and try new things, not being afraid of adventures, looking for new unconventional solutions) and agreeableness (the dimension characterized by positive attitudes towards others versus negative attitudes—being ready to sacrifice oneself for another person versus aversion and putting one’s own interests over others) of athletes are similar to those of non-training people [43,44,45,46,47].

In summary, we have proved a difference between sports level and personality in the raw score in openness to experience at the level of 0.049. The lack of such a difference in the sten score (openness to experience is at the same level of interpretation) indicates the specificity of American football. The obtained data confirm the significant impact of American football and its level on the shaping of the researched players’ personality. Sport activity (American football) shapes the players’ personality, and this is visible in the sten result—there is no difference in the obtained personality profiles. In turn, the shaped personality traits have an impact on making decisions in the starting situation at a given sports level, and this is visible as a raw result in the dimension of openness to experience. This is related to the specificity of sports rivalry in American football and slightly different psychological requirements that the players in the LFA 1, LFA 2, and LFA 9 leagues have.

In American football, we deal with a huge variety of tasks and sports techniques used. In addition, this sport is characterized by the accumulation of players with different personality profiles, which is why it was so important to generate personality profiles of American football players in Poland. It should be mentioned that similar studies were carried out in the homeland of American football, i.e., the United States. Schaubhut et al. [48] presented the results of the CPI 260^TM^ on 812 players who, in the years 2002–2005, applied for a professional contract in the best American football leagues in the world. It was shown that out of the studied group of quarterbacks, running backs, wide receivers, linebackers, kickers/punters, defensive backs, and defensive tackles, quarterbacks (QB) score higher on average than others on scales such as domination, independence, good impression, and leadership. In contrast, defensive players scored significantly lower than others on scales such as self-acceptance, social compliance, achievement through compliance, and work orientation. Interestingly, a comparison was also made between the group of players who, despite participating in the recruitment, did not receive an offer to play in any of the leagues, and the group of qualified footballers. The obtained results were remarkably similar to each other; however, the selected persons obtained, on average, slightly higher results on the self-control scale. Both research studies clearly showed how important it is to define personality in the recruitment of players to American football, which is aimed at acquiring players with the greatest sports potential that can be used in the long term [11]. In addition, we find it necessary to conduct further research on the relationship between personality and sports experience in all sports disciplines.

## 5. Conclusions

To summarize, openness to experience is a characteristic personality trait for American football players in Poland. Raw scores on this scale were the only ones that showed statistically significant differences between players representing the LFA 1, LFA 2, and LFA 9 league levels. It is recommended thus to systematically conduct personality tests among American football players in Poland in the process of recruiting candidates for football competition at the highest level. It can significantly contribute to a more accurate recruitment of players, and will also accelerate the development of the sports level of this discipline in Poland. In addition, the results of personality studies may prove useful in improving the predictions of important sporting behavior in American football, such as: individual results, reactions to failure, and long-term achievements. It should be noted that the sten results of American football players in Poland are at an average level, as are the data of Polish non-training persons. We assume that this proves the uniqueness of American football as a sports discipline for everyone. The raw results, on the other hand, show the influence of the level of the games on the shaping of the personality of American football players in Poland.

## Figures and Tables

**Table 1:** Values of descriptive statistics for the personality profile of American football players aged 20–29 years in Poland.

	M	SD	Min	Q25	Me	Q75	Max	Shapiro–Wilk Test (*p*)
Neuroticism (raw scores)	16.33	6.98	5.0	11.0	15.0	21.0	37.0	<0.001
Neuroticism (sten scores)	4.39	1.99	1.0	3.0	4.0	6.0	10.0	<0.001
Extraversion (raw scores)	33.01	7.38	14.0	29.0	34.0	39.0	46.0	<0.001
Extraversion (sten scores)	7.34	2.30	1.0	6.0	8.0	9.0	10.0	<0.001
Openness to experience (raw scores)	28.17	5.86	15.0	24.0	28.0	32.5	42.0	0.193
Openness to experience (sten scores)	5.61	1.85	1.0	4.0	6.0	7.0	10.0	0.001
Agreeableness (raw scores)	28.44	5.82	14.0	24.0	29.0	32.0	40.0	0.111
Agreeableness (sten scores)	5.61	2.12	1.0	4.0	5.0	7.0	10.0	0.001
Conscientiousness (raw scores)	34.84	7.50	15.0	30.0	36.0	41.0	48.0	0.001
Conscientiousness (sten scores)	7.09	2.22	1.0	6.0	7.0	9.0	10.0	<0.001

M—average; SD—standard deviation; Min—minimum value; Q25—lower quartile; Me—median; Q75—upper quartile; Max—maximum value; *p*—significance.

**Table 2:** Data on the personality profiles of American football players aged 20–29 years in Poland according to the LFA game level.

		M	SD	Min	Q25	Me	Q75	Max	Shapiro–Wilk Test Results (*p*)	Test Result
Neuroticism (raw scores)	LFA 1	16.79	7.11	5	12	15	21	36	0.001	H = 0.923 df = 2 *p* = 0.630
	LFA 2	16.00	6.91	7	11	15	19	37	0.005	
	LFA 9	15.48	6.86	6	10	14	21	34	0.090	
Neuroticism (sten scores)	LFA 1	4.48	2.02	1	3	4	6	10	0.006	H = 0.683 df = 2 *p* = 0.711
	LFA 2	4.38	1.96	2	3	4	6	10	0.007	
	LFA 9	4.12	2.01	1	2	4	6	9	0.061	
Extraversion (raw scores)	LFA 1	33.73	7.54	17	30	35	40	45	<0.001	H = 2.914 df = 2 *p* = 0.233
	LFA 2	32.83	6.80	15	30	34	38	46	0.300	
	LFA 9	31.16	7.74	14	26	33	37	44	0.160	
Extraversion (sten scores)	LFA 1	7.56	2.36	2	6	8	10	10	<0.001	H = 2.797 df = 2 *p* = 0.247
	LFA 2	7.30	2.08	2	7	8	9	10	0.004	
	LFA 9	6.72	2.44	1	5	7	9	10	0.056	
Openness to experience (raw scores)	LFA 1	29.29	5.45	18	26	29	33	42	0.298	F = 3.080 df1 = 2; df2 = 137 *p* = 0.049
	LFA 2	27.03	5.56	17	24	27	30	42	0.285	
	LFA 9	26.64	6.96	15	22	24	33	40	0.188	
Openness to experience (sten scores)	LFA 1	5.92	1.70	3	5	6	7	10	0.003	H = 4.850 df = 2 *p* = 0.088
	LFA 2	5.30	1.70	2	4	6	6	9	0.093	
	LFA 9	5.20	2.38	1	3	5	7	10	0.070	
Agreeableness (raw scores)	LFA 1	27.44	5.77	14	23	28	31	39	0.360	F = 2.417 df1 = 2; df2 = 137 *p* = 0.093
	LFA 2	29.53	5.60	16	26	29	35	39	0.323	
	LFA 9	29.68	6.00	19	25	31	33	40	0.487	
Agreeableness (sten scores)	LFA 1	5.28	2.07	1	4	5	7	10	0.016	H = 3.893 df = 2 *p* = 0.413
	LFA 2	5.98	2.13	2	5	6	8	10	0.267	
	LFA 9	6.04	2.17	3	4	6	8	10	0.064	
Conscientiousness (raw scores)	LFA 1	35.51	6.94	17	30	37	41	47	0.015	H = 1.363 df = 2 *p* = 0.506
	LFA 2	33.60	7.95	15	28	34	40	46	0.220	
	LFA 9	34.84	8.41	16	30	36	40	48	0.395	
Conscientiousness (sten scores)	LFA 1	7.35	2.06	2	6	8	9	10	<0.001	H = 2.343 df = 2 *p* = 0.310
	LFA 2	6.68	2.31	1	5	7	9	10	0.103	
	LFA 9	7.00	2.50	1	5	7	9	10	0.062	

M—average; SD—standard deviation; Min—minimum value; Q25—lower quartile; Me—median; Q75—upper quartile; Max—maximum value; p—significance; H—statistics of the Kruskal–Wallis test; F—ANOVA statistic; df—degrees of freedom.

## Data Availability

The authors confirm that the data supporting the findings of this study are available within the article.
